# Binocular vision findings in normally-sighted school aged children who used digital devices

**DOI:** 10.1371/journal.pone.0266068

**Published:** 2022-04-07

**Authors:** Urusha Maharjan, Sujata Rijal, Ashutosh Jnawali, Sanjeeta Sitaula, Sanjeev Bhattarai, Gulshan Bahadur Shrestha

**Affiliations:** 1 Drishti Eye Care System Hospital, Kathmandu, Nepal; 2 University of Houston, College of Optometry, Houston, Texas, United States of America; 3 New England College of Optometry, Boston, Massachusetts, United States of America; 4 Department of Ophthalmology, Maharajgunj Medical Campus, Tribhuvan University, Kathmandu, Nepal; Cairo University Kasr Alainy Faculty of Medicine, EGYPT

## Abstract

**Purpose:**

To determine the binocular vision status in normally-sighted school aged children who used digital devices.

**Methods:**

A cross-sectional study was conducted at B.P. Koirala Lions Center for Ophthalmic Studies, Kathmandu, Nepal for a duration of one year. One hundred and eighty school aged children (71 female and 109 male) aged 7 to 17 years were included in the study. All the children underwent detailed ophthalmic and binocular vision examinations. The duration of the use of digital devices by the children were asked to either the parents or guardians present at the time of the study. The study participants were divided into two groups: children who used digital devices for the last six months (users group) and those who hadn’t used digital devices for the last six months (non users group). The users group was again divided into two subgroups: children who used digital devices for less than 3 hours per day and a day per week (low digital device users subgroup) and children who used digital devices for more than 3 hours per day and all days in a week (high digital device users subgroup).

**Results:**

Accommodative amplitudes, accommodative facility, and positive fusional vergence for both near and distance were significantly reduced in the high digital device users group than in the low digital device users subgroup (p <0.01). Stereo acuity, near point of convergence, and negative fusional vergences for both near and distance were not statistically significantly different between the two subgroups. Prevalence of accommodative and vergence anomalies (except convergence insufficiency) was more in the high digital device users subgroup than in the low digital device users subgroup (p<0.01).

**Conclusions:**

Children who used digital devices for a significantly greater amount of time had significantly reduced amplitudes of accommodation, accommodative facility, and positive fusional vergence both at near and distance.

## Introduction

The use of digital devices (desktop, laptop and tablet computers, smartphones and electronic reading devices) have increased exponentially in recent years due to their necessity for both vocational and avocational purposes among all age groups [1, 2]. Parents also encourage children at very early years to use computers [3]. An investigation of over 2000 American children between 8 and 18 years of age found that, in an average day, they spent approximately 7.5 hours viewing entertainment media (comprising 4.5 hours watching television, 1.5 hours on a computer and over an hour playing computer games) [4]. Home or school computer access is also commonplace in many countries including urban and suburban areas of Nepal [5, 6].

With the rise in use of digital devices, there is increased demands for three dimensional perception, in particular stereopsis, and binocular single vision [7–10]. Binocular single vision requires coordinated use of both eyes, so that separate and slightly dissimilar images arising in each eye are appreciated as a single image by the process of fusion [11, 12]. For efficient single binocular vision to occur, the retinal images for the two eyes must be in good focus and of similar size and shape. This is promised by the good functioning of accommodative and vergence systems and also by the absence of any misalignment of the eyes and sensory anomalies.

The focusing mechanism in human eyes does not seem to work well with electronically generated characters as it works with printed characters that have well-defined edges with good background and contrast between the background and the letters [3, 13]. The characters on a computer screen are made of tiny dots called pixels. Each pixel is bright at its center and the brightness gradually decreases towards the outer edges. Therefore, electronically generated characters have blurred edges compared to letters on a printed page. Thus, the human eye finds it very difficult to maintain focus on pixel characters. This happens because when an attempt to focus on the plane of the computer is made, the eye fails to sustain the focus and relaxes to focus behind the screen [14]. This point is known as the resting point of accommodation or the dark focus. The eyes are therefore constantly relaxing to resting point of accommodation and straining to refocus on to the screen, ultimately leading to eye strain and fatigue [3, 15]. Thus, visual work on digital devices is challenging and includes frequent ocular motility, accommodation, and vergence demands [16]. The impairment of vergence and accommodative systems results in visual discomfort. This interferes significantly with visual attention, visual performance thereby limiting work capacity [17, 18].

Digital eye strain, commonly known as computer vision syndrome, is the combination of eye and vision problems associated with the use of computers and other electronic displays [7, 19]. It is a commonly observed syndrome in adults [20] but the concern is no less in children. Since adults have adopted and integrated digital devices in their lives, children have learned the same. This has led to children experiencing similar symptoms (such as eye ache, eye strain, double vision, lacrimation, burning eyes, blurring of vision, dryness and irritation) [21–23] related to computer use as by adults. However, when children use computers, they are more susceptible to developing problems than adults because of their unawareness towards the symptoms they are suffering, prolonged viewing without taking a break, or increased viewing angle when using digital devices set up for adults [2]. The visual discomfort caused by digital devices interferes with attention, academic performance, and limit ability to work [12, 17, 18]. It is reported that children with accommodative or vergence anomalies who use electronic devices for a significant amount of time have significantly lower GPA [21, 24].

Though it is suggested that the digital devices impact a child’s binocular single vision, only limited number of studies have been reported till date regarding it [25]. Various studies have addressed the subjective symptoms associated with the use of digital devices or have associated academic performance with the binocular vision findings (measuring the various parameters of binocular single vision) [21, 26]. However, only a few studies have correlated the refractive and binocular vision findings in school aged children who used digital devices [27, 28].

## Methods

The study was approved by the Institutional Review Committee of the Institute of Medicine, Tribhuvan University, Nepal, and adheres to the tenets of declarations of Helsinki. Informed verbal consent was taken from children and their parents or guardians who were present at the time of examination.

### Study design and subjects

This was a hospital based cross-sectional study conducted at B.P. Koirala Lions Center for Ophthalmic Studies (BPKLCOS), Institute of Medicine, Kathmandu, Nepal between December 2018 and November 2019. School aged children (n = 180), aged 7–16 years (mean age 13.01 ± 2.63 years), who had corrected monocular distance visual acuity of 6/6 with Snellen’s visual acuity chart were included. Subjects with any ophthalmic diseases, systemic diseases that are known to affect the eyes, or those with history of ocular surgery were excluded. A well illuminated room was used to carry out all the procedures and the subjects wore their habitual refractive correction (if present) during the procedures.

### Monocular visual acuity

Internally illuminated rotating drum with Snellen’s chart (Clement-Clarke Haag-Streit, Harlow, UK) was used to measure monocular visual acuity at 6 meters in a well illuminated room. Near chart (Roman test types, Drishti, Kathmandu, Nepal) was used to measure near visual acuity binocularly at 40 centimeters.

### Manifest distance refraction

Objective distance refraction was performed with a retinoscope (Heine Beta 200, Gilching, Germany) while the subject fixated at 6 meters with their accommodation relaxed. Following objective refraction, subjective refraction was performed to determine the final refraction for the subjects.

### Cover test

Cover/uncover test was done to assess any phoria or tropia if present. No movement on cover/uncover test was considered as orthophoria. Inward movement of the eye when cover was removed was considered as exophoria; whereas, outward movement was considered as esophoria [29]. Prism cover test was performed to measure the amount of phoria when present. Subjects with intermittent or manifest tropia were excluded from the study.

### Near stereoacuity

Titmus stereo test (stereo fly test, Stereo Optical company, Chicago, USA) was performed at 40 centimeters with the patient wearing polarized spectacles. Three sets of five animals is considered useful for young children [30] which was used to demonstrate and practice. The circles were then used to note the near stereoacuity.

### Near point of convergence

Royal air force rule (RAF, model ASF- ER300, India) was used to measure near point of convergence by push up method. Accommodative target (N5) was slowly pushed towards the subject at the recommended linear rate of about 1 ± 2 centimeters per second until subjects first noticed blur or diplopia. Three measurements were taken and the average was noted.

### Accommodative amplitude

Accommodative amplitude was measured using Royal air force rule by push up method. Near point of accommodation was measured by slowly towards the subject [31]. Subjective blur point was noted. It was repeated thrice and the average measurement was noted. The accommodative amplitude in centimeters was then converted to amplitude of accommodation in diopters by dividing it by 100 [32]. The procedure was done both monocularly and binocularly.

### Accommodative facility

Lens flipper (±2D) was used to measure the monocular and binocular accommodative facility with N8 target at 40 centimeters. All the participants had N6 or better near visual acuity, so they could fixate on the N8 line without any problem. Subjects were asked to read the letter when clear (were made to wait until the letter was clear with the lens) with the lenses and then flipped as soon as it got clear and they read out the letter. One cycle was noted when focusing through minus lens and plus lens was done. After a practice session of 30 seconds, monocular accommodative facility for right eye was noted and then repeated binocularly.

### Accommodative response

Monocular estimation method retinoscopy was performed on the right eye with the printed letters placed at 40 centimeters from the subject’s spectacle plane. Subjects were made to read the words and required lens power was quickly interposed to neutralize the movement observed by the retinoscope. The power of the neutralizing lens was noted.

### Fusional vergence

Horizontal prism bar was used to measure both negative and positive fusional vergences at near (40 centimeters) and distance (6 meters), assuming optical infinity. The prism bar was placed before the subject’s one eye and the power of prism was gradually increased. The test was stopped at the point of consistent diplopia or break. Positive fusional vergence was measured by placing base out prism and negative fusional vergence was measured by placing base in prism. Negative fusional vergence was measured before positive fusional vergence to avoid effect of convergence testing on vergence recovery [33].

### Questionnaire

A questionnaire asking the duration of digital devices used and the types of digital device was filled by parents/ guardians. These questionnaires were in English language. It was translated to Nepali language and asked by the optometrist to record the response, if necessary.

Digital devices consisted of desktop, laptop and tablet computers, televisions, and smartphones. All types of digital devices were placed in same category as children were using multiple devices throughout the day and it was not easy to separate one versus the others’ viewing time.

The participants were divided into two groups:

□ Non—users group (n = 10): children who hadn’t used digital devices for the last six months
□ Users group (n = 170): children who had used digital devices for the last six months

The users group was again divided into two subgroups:

▪ Low digital devices users (n = 87): children who used the digital devices for less than 3 hours per day and only a day per week
▪ High digital devices users (n = 83): children who used the digital devices for 3 hours or more than 3 hours per day and all days in a week

### Statistical analyses

- Independent samples t test was used to compare the means of the parameters of BSV among the two groups namely low digital device users subgroup and high digital device users subgroup.
- Paired samples t test was used to compare the means of near point of accommodation between the right and left eye of the subjects; and also for comparing the means of monocular accommodative facility between the right and left eye of the subjects. Since no significant difference was found between the two eyes, data from right eye only is used for comparing with the normative data.
- The Chi- square test was used to compare the prevalence of convergence insufficiency, accommodative insufficiency, accommodative infacility and fusional insufficiency among different groups of digital device users.
- One sampled z test was used to compare the binocular vision findings of present study with the normative data provided by the BAND study.
- *P-*value of less than 0.05 was considered statistically significant.

## Results

### Refractive error

Out of 180 subjects, majority of the children (n = 104) were emmetropic (± 0.25 Diopters). There were 42 myopic subjects (-0.50 to -2.00 Diopters), 21 subjects were astigmatic (-0.50 to -1.00 Diopters) and 13 subjects were hyperopic (+0.50 to +1.00 Diopters).

### Binocular vision findings of the non-users group

Since the number of children in the non-users group was significantly lower compared to the users group, the non-users group was not compared with the users group. The binocular vision findings of the non-users group were compared to the normative data taken from the BAND study [33] is shown in Table 1.

**Table 1:** Parameters of Binocular Vision (BSV) in non-users group.

Parameters of BSV in non-users group	Current Study	Normative data taken from the BAND study [33]	P-value
Stereo acuity	51 ± 15.95 seconds of arc	40 ± 15 seconds of arc	0.20
Monocular NPA (RE) (in centimeters)	7–10 years: 6.50 ± 0.58	7–10 years: 13 ± 3	<0.01*
	11–17 years: 6.40 ± 0.55	11–17 years: 11 ± 2	<0.01*
Binocular NPA (in centimeters)	7–10 years: 6.50 ± 0.58	7–10 years: 13 ± 3	<0.01*
	11–17 years: 6.40 ± 0.55	11–17 years: 11 ± 3	<0.01*
MAF (RE) (cycles per minute)	7–12 years: 12.08 ± 1.79	7–12 years: 11 ± 4	0.55
	13–17 years: 9.40 ± 2.33	13–17 years: 14 ± 5	0.04*
BAF (cycles per minute)	7–12 years: 12.30 ± 2.33	7–12 years: 10 ± 4	0.20
	13–17 years: 9.00 ± 3.08	13–17 years: 14 ± 5	0.03*
Lag of Accommodation	0.13 ± 0.13 Diopters	0.4 ± 0.2 Diopters	<0.01*
NPC (in centimeters)	7.00 ± 1.41	3 ± 3	<0.01*
PFV for distance (prism diopters)	15.00 ± 4.24	17 ± 8	0.43
PFV for near (prism diopters)	25.00 ± 7.07	26 ± 10	0.75
NFV for distance (prism diopters)	9.14 ± 0.74	8 ± 2	0.71
NFV for near (prism diopters)	19.00 ± 1.41	15 ± 4	<0.01*

BAF = Binocular Accommodative Facility, LE = left eye, MAF = Monocular Accommodative Facility, NFV = Negative Fusional Vergence, NPA = Near Point of Accommodation, NPC = Near Point of Convergence, PFV = Positive Fusional Vergence, RE = right eye

### Comparison of binocular vision findings of low digital device users subgroup with the normative data taken from the BAND study

In our study, Table 2 shows the comparison of our findings with the normative data given by the BAND study [34].

**Table 2:** Comparison of binocular vision findings of low digital device users subgroup with the normative data taken from the BAND study.

Parameters of BSV	Low digital device users subgroup (n = 87)	Normative data Taken from the BAND study [33]	*P-* value
Stereo acuity	52.35 ± 31.25 seconds of arc	40±15 seconds of arc	<0.01*
Monocular NPA (RE) (in centimeters)	7–10 years: 6.56 ± 0.78	7–10 years: 13 ± 3	<0.01*
	11–17 years: 6.96 ± 1.50	11–17 years: 11 ± 2	<0.01*
Binocular NPA (in centimeters)	7–10 years: 6.56 ± 0.78	7–10 years: 13 ± 3	<0.01*
	11–17 years: 6.69 ± 1.12	11–17 years: 11 ± 3	<0.01*
MAF (RE) (cycles per minute)	7–12 years: 10.62 ± 2.63	7–12 years: 11 ± 4	0.57
	13–17 years: 10.91 ± 3.52	13–17 years: 14 ± 5	<0.01*
BAF (cycles per minute)	7–12 years: 9.33 ± 2.49	7–12 years: 10 ± 4	0.32
	13–17 years: 10.07 ± 3.50	13–17 years: 14 ± 5	<0.01*
Lag of Accommodation (diopters)	0.43 ± 0.40	0.4 ± 0.2	1.62
NPC (centimeters)	7.08 ± 2.39	3 ± 3	<0.01*
PFV for distance (prism diopters)	16.01 ± 7.17	17 ± 8	0.25
PFV for near (prism diopters)	31.00 ± 10.89	26 ± 10	<0.01*
NFV for distance (prism diopters)	9.48 ± 3.55	8 ± 2	<0.01*
NFV for near (prism diopters)	14.19 ± 4.29	15 ± 4	0.06

* = p value less than 0.01.

Abbreviations: BAF = Binocular Accommodative Facility, LE = left eye, MAF = Monocular Accommodative Facility, NFV = Negative Fusional Vergence, NPA = Near Point of Accommodation, NPC = Near Point of Convergence, PFV = Positive Fusional Vergence, RE = right eye.

### Comparison of binocular vision findings of high digital device users subgroup with the normative data taken from the BAND study

In our study, Table 3 shows the comparison of our findings with the normative data given by the BAND study [34].

**Table 3:** Comparison of high digital device users subgroup with the normative data taken from the BAND study.

Parameters of BSV	High digital device users subgroup (n = 83)	Normative data taken from the BAND study [33]	*P -*value
Stereo acuity	46.75 ± 14.21 seconds of arc	40±15 seconds of arc	<0.01*
Monocular NPA (RE) (in centimeters)	7–10 years: 7.29 ± 1.49	7–10 years: 13 ± 3	<0.01*
	11–17 years: 7.69 ± 1.98	11–17 years: 11 ± 2	<0.01*
Binocular NPA (in centimeters)	7–10 years: 7.21 ± 1.53	7–10 years: 13 ± 3	<0.01*
	11–17 years: 7.64 ± 1.94	11–17 years: 11 ± 3	<0.01*
MAF (RE) (cycles per minute)	7–12 years: 6.88 ± 3.57	7–12 years: 11 ± 4	<0.01*
	13–17 years: 8.52 ± 3.36	13–17 years: 14 ± 5	<0.01*
BAF (cycles per minute)	7–12 years: 6.76 ± 2.77	7–12 years: 10 ± 4	<0.01*
	13–17 years: 8.20 ± 3.17	13–17 years: 14 ± 5	<0.01*
Lag of Accommodation (diopters)	0.81 ± 0.26	0.4 ± 0.2	<0.01*
NPC (centimeters)	7.15 ± 2.43	3 ± 3	<0.01*
PFV for distance (prism diopters)	12.31 ± 6.30	17 ± 8	<0.01*
PFV for near (prism diopters)	21.87 ± 9.03	26 ± 10	<0.01*
NFV for distance (prism diopters)	8.59 ± 3.75	8 ± 2	<0.01*
NFV for near (prism diopters)	14.12 ± 5.36	15 ± 4	0.05

Abbreviations: BAF = Binocular Accommodative Facility, LE = left eye, MAF = Monocular Accommodative Facility, NFV = Negative Fusional Vergence, NPA = Near Point of Accommodation, NPC = Near Point of Convergence, PFV = Positive Fusional Vergence, RE = right eye

* = p value less than 0.01.

### Binocular vision findings of the users group

Table 4 represents the binocular vision findings of the users group which was divided into two subgroups, low and high digital device users subgroups. In our study, we compared our findings with the normative data given by the BAND study [34].

**Table 4:** Binocular vision findings between low and high digital device users subgroup.

Parameters of BSV	Low digital device users subgroup (n = 87)	High digital device users subgroup (n = 83)	*P-value*
Stereo acuity	52.35 ± 31.25 seconds of arc	46.75 ± 14.21 seconds of arc	0.15
Monocular NPA (RE) (in centimeters)	7–10 years: 6.56 ± 0.78	7–10 years: 7.29 ± 1.49	0.46
	11–17 years: 6.96 ± 1.50	11–17 years: 7.69 ± 1.98	<0.01*
Binocular NPA (in centimeters)	7–10 years: 6.56 ± 0.78	7–10 years: 7.21 ± 1.53	0.45
	11–17 years: 6.69 ± 1.12	11–17 years: 7.64 ± 1.94	<0.01*
MAF (RE) (cycles per minute)	7–12 years: 10.62 ± 2.63	7–12 years: 6.88 ± 3.57	<0.01*
	13–17 years: 10.91 ± 3.52	13–17 years: 8.52 ± 3.36	<0.01*
BAF (cycles per minute)	7–12 years: 9.33 ± 2.49	7–12 years: 6.76 ± 2.77	<0.01*
	13–17 years: 10.07 ± 3.50	13–17 years: 8.20 ± 3.17	0.02*
Lag of Accommodation (diopters)	0.43 ± 0.40	0.81 ± 0.26	0.03*
NPC (centimeters)	7.08 ± 2.39	7.15 ± 2.43	0.99
PFV for distance (prism diopters)	16.01 ± 7.17	12.31 ± 6.30	<0.01*
PFV for near (prism diopters)	31.00 ± 10.89	21.87 ± 9.03	<0.01*
NFV for distance (prism diopters)	9.48 ± 3.55	8.59 ± 3.75	0.22
NFV for near (prism diopters)	14.19 ± 4.29	14.12 ± 5.36	0.94

*p value less than 0.05.

Abbreviations: BAF = Binocular Accommodative Facility, LE = left eye, MAF = Monocular Accommodative Facility, NFV = Negative Fusional Vergence, NPA = Near Point of Accommodation, NPC = Near Point of Convergence, PFV = Positive Fusional Vergence, RE = right eye.

### Comparison of prevalence of accommodative and vergence anomalies between the low and high digital device user subgroups

Among the 180 subjects in the present study, 51 subjects had combined accommodative and vergence anomalies and 56 subjects had normal binocular vision findings. The prevalence of accommodative and vergence anomalies between the low and high digital device users subgroups when compared to each other showed that the prevalence was significantly different in terms of accommodative infacility (AIF), accommodative insufficiency (AI) and fusional insufficiency (FI). But there was no significant difference between the two subgroups in terms of convergence insufficiency as shown in Table 5.

**Table 5:** Comparison of prevalence of accommodative and vergence anomalies between low and high digital device users subgroups.

Type of anomaly	Frequency in low digital device users subgroup (n = 86)	Frequency in high digital device users subgroup (n = 82)	P value (Chi square)
AIF	25 (29.07%)	55 (67.10%)	0.002*
AI	14 (16.28%)	26 (31.71%)	0.004*
CI	16 (18.60%)	26 (31.71%)	0.075
FI	43 (50%)	59 (71.95%)	0.006*

*p value less than 0.01

Abbreviations: AI = Accommodative Insufficiency, AIF = Accommodative Infacility, CI = Convergence Insufficiency, FI = Fusional Insufficiency

(NOTE: The above mentioned frequencies are single count; thus, the total will not add up to total number of users group)

## Discussion

Digital devices have been applied in an increasing amount of work recently [22]. Many studies have speculated that rising prevalence of asthenopia in the young is due to increased use of mobile phones [1, 22, 23, 26]. However, most of the studies [21, 26] have subjectively assessed the various vision related symptoms (such as blurred vision, double vision, etc.) experienced with increased use of digital devices and doesn’t include the changes in the binocular vision with the use of digital devices. This study has associated the binocular vision findings of children using digital devices and showed adverse impact on accommodative amplitude, accommodative facility and positive fusional vergences with the increased use of digital devices.

In this study, there were only 10 non usersand 170 digital device users. Users group was divided into low and high digital device users subgroup. This study compared the binocular vision findings with the normative data provided by BAND study done in Tamil Nadu, India [35]. Since there were only 10 subjects in the non users group, the binocular vision findings of the non users group was not compared with the users group. The binocular vision findings were within normal limits in the non users group when compared to the normative data as shown in Table 1. However, due to the limited data from the non-users group, the comparison is unreliable.

The mean stereo acuity between the two subgroups of users were compared with the normative data taken from the BAND study and it showed significantly reduced stereoacuity in both low and high digital device users subgroup as shown in Tables 3 and 4. This is similar to the findings of the study by Rechichi et al where the video game group had significantly lower percentage of stereo acuity when compared to the non users group of their study [1]. The accommodative amplitude was significantly better than the normative data taken from the BAND study as shown in Tables 3 and 4; however, the accommodative amplitude was reduced in the high digital device users subgroup than in the low digital device users subgroup as shown in Table 4; The presence of accommodative insufficiency was more in the high digital device users subgroup (31.71%) than in the low digital device users subgroup (16.28%) as shown in Table 5. This finding of low digital device users subgroup is twice as greater as that of Shrestha et al.’s findings (9.7%) [29] and almost equal to the findings of Davis et al. [36] However, our study’s finding was in contrast to the findings by Amit et al. [28] where there was no accommodation insufficiency in a group of children using digital devices for more than 4 hours per day. Most parents claimed that their children had a habit of using digital devices while doing their homework. The constant change of focus from the digital screen to their required homework copies needs good accommodative system. Prevalence of accommodative insufficiency as high as our findings is alarming to the caretakers of the children at school and at home [37].

Current study suggested that the monocular accommodative facility is decreased with the increased use of the digital devices in school aged children as shown in Table 4. But even the mean monocular accommodative facility of the low digital device users subgroup for age range 13–17 was significantly lower than the normative range as shown in Table 2. This points that the monocular accommodative facility is significantly reduced by the use of digital devices for a considerable amount of time.

Likewise, the mean binocular accommodative facility in the two subgroups showed significant differences as shown in Table 4. It suggests that with the increased use of digital devices, supposedly more than 3 hours all days in a week, would reduce the binocular accommodative facility in school aged children. In addition, when the low digital device users subgroup of age range 13–17 was compared with the normative data as shown in Table 2, it was lower than the normal range.

The prevalence of accommodative infacility, measured binocularly, was 35.5% in a study by Shrestha et al. [29] In contrast to that, our study showed a prevalence of 67.10% in the high digital device users subgroup, as shown in Table 5, which is almost as twice. This suggests the increment in the presence of accommodative problems in the recent days even in school aged children group.

Current study showed no significant differences in the mean near point of convergence between the two subgroups as shown in Table 4. This was in contrast to Krasina et al.’s [38] finding that the increased use of digital devices could impact the convergence system. The insignificant differences between the two subgroups in terms of near point of convergence could not explain the impact of use of digital device on vergence system but this might be due to the fact that phoria at near and distance was not considered to classify the patient with convergence insufficiency.

The prevalence of convergence insufficiency was 18.60% in the low digital device users subgroup and was 31.71% in the high digital device users subgroup. There was no statistically significant difference between the prevalence of convergence insufficiency in the two subgroups as shown in Table 5. Our findings are in support with the findings of Nyman et al. [39] where no significant differences was established between the video display terminal operators and the referents in terms of convergence capacity.

Our study found significant differences between the means of positive fusional vergence (sometimes referred to as amplitude of convergence) for both near and distance between the low digital device users subgroup and high digital device users subgroup. The mean positive fusional vergence of the low digital device users subgroup at distance and near was lower than the normative data. This suggests that the use of digital devices reduces the range of positive fusional vergence, which gets worse with the increased use for a significant amount of time.

In this current study, the presence of fusional insufficiency (reduced positive fusional vergence) was 50% in the low digital device user subgroup and was 71.95% in the high digital device use subgroup. These values are very high when compared to the findings of Shrestha et al [29] (which was 14.8%). This suggests that maybe the use of digital devices by school aged children is increasing day by day and by the same amount, the prevalence of fusional insufficiency is increasing too.

Although significant difference was established in terms of positive fusional vergence, the findings of negative fusional vergence for both near and distance between the two subgroups were not statistically significant as shown in Table 4. The findings were not reduced than the normative range.

### Limitations of the study

Unlike other studies, which mainly focuses on the subjective symptoms [21, 22, 26] or focuses on the adult population [20, 29], this study included school aged children who were exposed to digital devices. The limitations of this study was that it didn’t include any assessment of subjective symptoms experienced by the subjects. So, the quantification of their asthenopic symptoms were not addressed in this study. Present study evaluated the objective findings that could be the underlying cause of visual problems associated with increased usage of digital devices by children population.

### Conclusions

Current study suggested that higher durations of exposure to digital devices (for more than three hours a day and all days a week) among school aged children from 7 to 17 years of age might be associated with various eye and vision related problems.

The findings of this study showed that the parameters of binocular vision like accommodative facility, accommodative amplitude and positive fusional vergences are reduced with the increased exposure to digital device. This suggests inability to concentrate for longer periods during near visual work which can reduce the level of student achievement [10, 21].

## Supporting information

S1 File (DOCX)

## References

[1] Rechichi C, Aragona P. Video Game Vision Syndrome: A New Clinical Picture in Children? J Pediatr Ophthalmol Strabismus. 2017;54(6):346–55. doi: 10.3928/01913913-20170510-01 28850642

[2] Kozeis N. Impact of computer use on children’s vision. Hippokratia. 2009;13:230–1. 20011087 PMC2776336

[3] Saman Wimalasundera. Computer Vision Syndrome. Gall Med J.2009,11(1):25–9.

[4] Rosenfield M, Howarth PA, Sheedy JE, Crossland MD. Vision and IT displays: A whole new visual world. Ophthalmic Physiol Opt [Internet]. 2012 Sep [cited 2019 Nov 22];32(5):363–6. doi: 10.1111/j.1475-1313.2012.00936.x 22882149

[5] Acharya S, Sinnott L. Internet usage of teenagers in Nepal for educational purposes An analysis of Internet usage behaviour of 15-17-year-old students at selected schools in Kathmandu [Internet]. 2016. Available from: http://www.centrum3.at/fileadmin/downloads/VWA/Acharya_Internet_Nepal.pdf.

[6] Giri B, Dhakal MR. Utilization of Available Facilities of Computer Aided Teaching in Secondary Level School of Nepal. J Adv Acad Res. 2018;4(2):110–8.

[7] Rosenfield M. Computer vision syndrome (a.k.a. digital eye strain). Optom Pract. 2016;17(1):1–10.

[8] Sheppard AL, Wolffsohn JS. Digital eye strain: Prevalence, measurement and amelioration. BMJ Open Ophthalmol. 2018;3(1). doi: 10.1136/bmjophth-2018-000146 29963645 PMC6020759

[9] Wajuihian SO. Frequency of asthenopia and its association with refractive errors. African Vis Eye Heal. 2015;74(1):293–9.

[10] Nunes AF, Monteiro PML, Ferreira FBP, Nunes AS. Convergence insufficiency and accommodative insufficiency in children. BMC Ophthalmol. 2019;19(1):1–8. doi: 10.1186/s12886-018-1008-7 30791877 PMC6385397

[11] Bhola R. EyeRounds.org:[Internet]. Ophthalmology and Visual Sciences. 2006. Available from: https://webeye.ophth.uiowa.edu/eyeforum/tutorials/Bhola-BinocularVision.htm.

[12] O’Connor AR, Birch EE, Anderson S, Draper H, Group the FR. The Functional Significance of Stereopsis. Invest Ophthalmol Vis Sci. 2010 Apr 1;51(4):2019–23. doi: 10.1167/iovs.09-4434 19933184

[13] Mulik SS. A Critical review on Computer Vision Syndrome and its Ayurvedic Approach. Asian J Multidiscip Stud. 2015;3(3):128–31.

[14] Akinbinu TR, Mashalla YJ. Medical Practice and Review Impact of computer technology on health: Computer Vision Syndrome (CVS). Med Pract Rev. 2014;5(November):20–30.

[15] Yan Z, Hu L, Chen H, Lu F. Computer Vision Syndrome: A widely spreading but largely unknown epidemic among computer users. Comput Human Behav. 2008;24(5):2026–42.

[16] Jiménez R, Pérez MA, García JA, González MD. Statistical normal values of visual parameters that characterize binocular function in children. Ophthalmic Physiol Opt. 2004;24(6):528–42. doi: 10.1111/j.1475-1313.2004.00234.x 15491481

[17] Gowrisankaran S, Sheedy JE. Computer vision syndrome: A review. Work. 2015;52(2):303–14. doi: 10.3233/WOR-152162 26519133

[18] Xu Y, Deng G, Wang W, Xiong S, Xu X. Correlation between handheld digital device use and asthenopia in Chinese college students: a Shanghai study. Acta Ophthalmol. 2019;97(3):e442–7. doi: 10.1111/aos.13885 30272832

[19] Rosenfield M. Computer vision syndrome: A review of ocular causes and potential treatments. Ophthalmic Physiol Opt. 2011;31(5):502–15. doi: 10.1111/j.1475-1313.2011.00834.x 21480937

[20] Kharel Sitaula R, Khatri A. Knowledge, Attitude and practice of Computer Vision Syndrome among medical students and its impact on ocular morbidity. J Nepal Health Res Counc. 2018;16(3):291–6. 30455488

[21] Paudel N, Gyawali R, Pokharel R, Shrestha B, Loughman J. Does digital device usage affect children’s visual health and academic performance? 2019 Oct 24; doi: 10.13140/RG.2.2.11434.16321

[22] Moon JH, Lee MY, Moon NJ. Association between video display terminal use and dry eye disease in school children. J Pediatr Ophthalmol Strabismus. 2014;51(2):87–92. doi: 10.3928/01913913-20140128-01 24495620

[23] Moon JH, Kim KW, Moon NJ. Smartphone use is a risk factor for pediatric dry eye disease according to region and age: A case control study Pediatrics and Strabismus. BMC Ophthalmol. 2016;16(1). 10.1186/s12886-016-0364-4 PMC5084437 27788672

[24] Hawi NS, Samaha M. To excel or not to excel: Strong evidence on the adverse effect of smartphone addiction on academic performance. Comput Educ. 2016;98:81–9.

[25] Jaiswal S, Asper L, Long J, Lee A, Harrison K, Golebiowski B. Ocular and visual discomfort associated with smartphones, tablets and computers: what we do and do not know. Clin Exp Optom. 2019;102(5):463–77. doi: 10.1111/cxo.12851 30663136

[26] Ichhpujani P, Singh RB, Foulsham W, Thakur S, Lamba AS. Visual implications of digital device usage in school children: A cross-sectional study. BMC Ophthalmol. 2019;19(1):1–8. doi: 10.1186/s12886-018-1008-7 30866885 PMC6417240

[27] Mohan A, Sen P, Shah C, Jain E, Jain S. Prevalence and risk factor assessment of digital eye strain among children using online e-learning during the COVID-19 pandemic: Digital eye strain among kids (DESK study-1). Indian J Ophthalmol. 2021 Jan 1;69(1):140. doi: 10.4103/ijo.IJO_2535_20 33323599 PMC7926141

[28] Mohan A, Sen P, Shah C, Datt K, Jain E. Binocular accommodation and vergence dysfunction in children attending online classes during the COVID-19 pandemic: digital eye strain in kids (DESK) study-2. J Pediatr Ophthalmol Strabismus. 2021 Jul 1;58(4):224–35. doi: 10.3928/01913913-20210217-02 34288760

[29] Shrestha GS, Mohamed FN, Shaha DN. Visual problems among video display terminal (VDT) users in Nepal. J Optom. 2011;4(2):56–62.

[30] Li S, Zou H, Wei C. Stereoscopic visual acuity in types of ametropic amblyopia in children. J Pediatr Ophthalmol Strabismus. 2014;51(2):105–10. doi: 10.3928/01913913-20140220-04 24804304

[31] Wajuihian SO, Hansraj R. Vergence anomalies in a sample of high school students in South Africa. J Optom. 2016;9(4):246–57. doi: 10.1016/j.optom.2015.10.006 26750804 PMC5030317

[32] Hashemi H, Khabazkhoob M, Nabovati P, Shahraki FA, Ostadimoghaddam H, Faghihi M, et al. Accommodative insufficiency in a student population in Iran. J Optom. 2019 Jul 1;12(3):161–7. doi: 10.1016/j.optom.2018.03.006 29802027 PMC6612034

[33] Hussaindeen JR, Rakshit A, Singh NK, Swaminathan M, George R, Kapur S, et al. Binocular vision anomalies and normative data (BAND) in Tamil Nadu: report 1. Clin Exp Optom. 2017;100(3):278–84. doi: 10.1111/cxo.12475 27796049

[34] Hussaindeen JR, Rakshit A, Singh NK, George R, Swaminathan M, Kapur S, et al. Prevalence of non-strabismic anomalies of binocular vision in Tamil Nadu: report 2 of BAND study. Clin Exp Optom. 2017 Nov 1;100(6):642–8. doi: 10.1111/cxo.12496 27859646

[35] Hussaindeen JR, Rakshit A, Singh NK, Swaminathan M, George R, Kapur S, et al. Binocular vision anomalies and normative data (BAND) in Tamil Nadu: report 1. Clin Exp Optom. 2017;100(3):278–84. doi: 10.1111/cxo.12475 27796049

[36] Davis AL, Harvey EM, Twelker JD, Miller JM, Leonard-Green T, Campus I. Convergence Insufficiency, Accommodative Insufficiency, Visual Symptoms, and Astigmatism in Tohono O’odham Students. J Ophthalmol. 2016;2016. 10.1155/2016/6963976 PMC4971328 27525112

[37] Hussaindeen JR, Murali A. Accommodative insufficiency: Prevalence, impact and treatment options. Clin Optom. 2020;12:135–49. doi: 10.2147/OPTO.S224216 32982529 PMC7494425

[38] Valcheva KP, Krivoshiiska-Valcheva EK, Stateva D V., Statev KN. Computer Eye Syndrome in Children Aged 3 To 6 Years. J IMAB—Annu Proceeding (Scientific Pap. 2016;22(1):1075–7.

[39] Nyman KG, Knave BG, Voss M. Work with video display terminals among office employees. IV. Refractions, accommodation, convergence and binocular vision. Scand J Work Environ Heal. 1985;11(6):483–7. 10.5271/sjweh.2198 4095526

