# Supplementary material for: Binocular vision findings in normally-sighted school aged children who used digital devices
Source: PLoS One. 2022 Apr 7;17(4):e0266068. doi: 10.1371/journal.pone.0266068 (PMC8989299; doi:10.1371/journal.pone.0266068)
Supplement: S1 File — (DOCX) [file pone.0266068.s001.docx]

**Supporting information**

**Questionnaire**

**BINOCULAR VISION FINDINGS IN NORMALLY-SIGHTED SCHOOL AGED CHILDREN WHO USED DIGITAL DEVICES**

Name:

Age/Sex:

File No. :

Grade:

Address:

Contact No.:

Use of digital devices: Yes/No

Use of digital devices per day: …….. (in hours)

Use of digital devices per week: …… (in days)

| **Visual Acuity** | Right eye | Left eye |
| --- | --- | --- |
| 1. Unaided |  |  |
| 1. Aided |  |  |
| 1. Pinhole |  |  |

**Refraction**

|  | Sphere | Cylinder | Axis | Visual acuity | Spherical Equivalent |
| --- | --- | --- | --- | --- | --- |
| Right Eye |  |  |  |  |  |
| Left Eye |  |  |  |  |  |

**Titmus Fly Test (stereo acuity):** ……… Seconds of arc

**Near Point of Convergence:** ……… centimeters

**Near Point of Accommodation:**

1. Monocular (Right eye): …… centimeters
2. Monocular (Left eye): ……. centimeters
3. Binocular: ……. centimeters

**Dynamic Retinoscopy Findings:** ………….

**Flipper ±2D test:**

1. Monocular (Right Eye): ….. cycles per minute
2. Monocular (Left Eye): …... cycles per minute
3. Binocular: …… cycles per minute

**Prism Fusion Range:**

|  | Positive Fusional Vergence | Negative Fusional Vergence |
| --- | --- | --- |
| At 6 meters | …….. prism diopters | ……. prism diopters |
| At 40 centimeters | …….. prism diopters | ……. prism diopters |
